# Changes in the Gut Microbiome and Predicted Functional Metabolic Effects in an Australian Parkinson’s Disease Cohort

**DOI:** 10.3389/fnins.2021.756951

**Published:** 2021-10-29

**Authors:** Jade E. Kenna, Eng Guan Chua, Megan Bakeberg, Alfred Tay, Sarah McGregor, Anastazja Gorecki, Malcolm Horne, Barry Marshall, Frank L. Mastaglia, Ryan S. Anderton

**Affiliations:** ^1^School of Medicine, The University of Western Australia, Nedlands, WA, Australia; ^2^Centre for Neuromuscular and Neurological Disorders, The University of Western Australia, Nedlands, WA, Australia; ^3^Centre for Clinical Neurosciences and Neurological Research, St. Vincent’s Hospital Melbourne, Fitzroy, VIC, Australia; ^4^Perron Institute for Neurological and Translational Science, Nedlands, WA, Australia; ^5^School of Biological Sciences, The University of Western Australia, Crawley, WA, Australia; ^6^Marshall Centre for Infectious Diseases Research and Training, The University of Western Australia, Nedlands, WA, Australia; ^7^School of Medicine, University of Notre Dame Australia, Fremantle, WA, Australia; ^8^Florey Institute of Neuroscience and Mental Health, The University of Melbourne, Parkville, VIC, Australia; ^9^Institute for Health Research, University of Notre Dame Australia, Fremantle, WA, Australia; ^10^School of Nursing, Midwifery, Health Sciences and Physiotherapy, The University of Notre Dame Australia, Fremantle, WA, Australia

**Keywords:** Parkinson’s disease, gut microbiome, 16S, gut bacteria, KEGG

## Abstract

**Background:** There has been increasing recognition of the importance of the gut microbiome in Parkinson’s disease (PD), but the influence of geographic location has received little attention. The present study characterized the gut microbiota and associated changes in host metabolic pathways in an Australian cohort of people with PD (PwP).

**Methods:** The study involved recruitment and assessment of 87 PwP from multiple Movement Disorders Clinics in Australia and 47 healthy controls. Illumina sequencing of the V3 and V4 regions of the 16S rRNA gene was used to distinguish inter-cohort differences in gut microbiota; KEGG analysis was subsequently performed to predict functional changes in host metabolic pathways.

**Results:** The current findings identified significant differences in relative abundance and diversity of microbial operational taxonomic units (OTUs), and specific bacterial taxa between PwP and control groups. Alpha diversity was significantly reduced in PwP when compared to controls. Differences were found in two phyla (Synergistetes and Proteobacteria; both increased in PwP), and five genera (C*olidextribacter, Intestinibacter, Kineothrix, Agathobaculum*, and *Roseburia*; all decreased in PwP). Within the PD cohort, there was no association identified between microbial composition and gender, constipation or use of gastrointestinal medication. Furthermore, KEGG analysis identified 15 upregulated and 11 downregulated metabolic pathways which were predicted to be significantly altered in PwP.

**Conclusion:** This study provides the first comprehensive characterization of the gut microbiome and predicted functional metabolic effects in a southern hemisphere PD population, further exploring the possible mechanisms whereby the gut microbiota may exert their influence on this disease, and providing evidence for the incorporation of such data in future individualized therapeutic strategies.

## Introduction

Parkinson’s disease (PD) is a debilitating neurodegenerative disorder thought to be caused by a combination of genetic and environmental factors. Although the presence of motor impairments primarily determine the diagnosis of PD (DeMaagd and Philip, 2015), a range of non-motor symptoms including impaired olfaction and gastrointestinal (GI) dysfunction may be present at the time of diagnosis, or even before the onset of motor manifestations. There is some evidence that some GI symptoms such as constipation may appear even decades before the development of the classic motor symptoms resulting from nigrostriatal dopaminergic neuronal death, suggesting the involvement of peripheral systems in the initial development and primary stages of PD. Given the interface between environmental factors and the GI tract, and its potential role in prodromal symptoms of the disease, research has increasingly focused on the gut microbiota as a potential regulator in the origin and pathogenesis of PD.

Evidence is accumulating of a bidirectional communication between the gut microbiome and the nervous system, allowing the modulation of brain activity, immunological function, and behavior based on the gut’s bacterial composition (Martin et al., 2018). Indeed, there is evidence that an unbalanced pro-inflammatory (“dysbiotic”) microbiota may adversely affect the host, resulting in disease (Schippa and Conte, 2014). Disruptions to the balanced ecosystem of the gut microbiota can result in increased inflammation in the GI tract, commonly presenting as GI symptoms. These disruptions may be triggered by factors including poor diet and exercise patterns, and chemical and pesticide exposure, which may increase the risk of developing PD (Nandipati and Litvan, 2016). Furthermore, a positive relationship between inflammatory bowel diseases (such as Ulcerative Colitis and Crohn’s disease) and PD risk has been reported in a number of populations, which are usually accompanied by proinflammatory changes to the gut microbiota (Lai et al., 2019; Villumsen et al., 2019). In response to microbial dysbiosis, disrupted intestinal barrier permeability and signaling may contribute to immune activation the formation of insoluble α-synuclein aggregates, a key pathological feature of PD.

It has been proposed that such α-synuclein aggregation first occurs in the peripheral and enteric nervous systems before spreading to the brain. Such retrograde spread of insoluble α-synuclein aggregates via the gut-brain axis has been previously been demonstrated in rodent models (Challis et al., 2020), and aggregation in these systems has been shown to associate with increased intestinal permeability and inflammation (Braak et al., 2003, 2006; Forsyth et al., 2011; Gorecki et al., 2019). Taken together this evidence raises the possibility that individuals who develop PD may undergo a proinflammatory shift in microbiota composition during the prodromal phases of developing the disease, coinciding with the earliest symptoms and pathological hallmarks appearing in the GI tract.

It is well established that the composition of the gut microbiome varies according to geographic location (Gaulke and Sharpton, 2018). However, as the influence of geographic location on the gut microbiome in PD has not been comprehensively investigated, it remains unknown whether some of the reported differences in the microbiome of affected individuals could be attributable to geographic factors, or are a commonality of the disease itself. To this end, comparative studies of the microbiome in people with Parkinson’s disease (PwP) from different geographical populations are of interest, in order to help solidify the knowledge surrounding the relationship between the gut microbiome and PD. So far, there have been only a few studies from the Southern hemisphere. A previous pilot study in a small PD cohort from our laboratory found that the relative abundance of mucin-degrading Verrucomicrobia and lipopolysaccharide-producing Gammaproteobacteria groups were increased in PwP (Gorecki et al., 2019). Recently, a comparative study of the gut microbiome in small groups of PwP undergoing device-assisted therapies has also been reported (Lubomski et al., 2021). In the Northern hemisphere, studies have documented significantly altered levels of various bacterial taxa, predominantly a decrease in the family Bacteroidetes (Prevotellaceae), and Phyla Firmicutes (Clostridiaceae, clostridium and butyricicoccus spp.; Lachnospiraceae, Blautia, dorea, roseburia, and coprococcus spp.,; Ruminococcaceae, *Faecalibacterium* spp.) (Keshavarzian et al., 2015; Bedarf et al., 2017; Petrov et al., 2017; Heintz-Buschart et al., 2018; Cirstea et al., 2020). Together, the reduction in these bacterial taxa and shift in microbiome composition are hypothesized to be a cause of increased intestinal permeability and inflammation, leading to the prodromal GI symptoms reported in the majority of PwP.

In this more comprehensive study we have investigated the role of the gut microbiome in PD by combining 16S rRNA sequencing and functional predictions of alterations in host metabolic pathways in a larger multicentre Australian cohort.

## Materials and Methods

### Study Participants

This study involved 87 idiopathic PwP recruited from the Movement Disorders Clinics at the Perron Institute for Neurological and Translational Science (Perth and Albany, Australia) and St Vincent’s Hospital (Melbourne, Australia), and 47 controls without PD who comprised spouses and volunteers in the same approximate age range who had no prior history of a neurological disorder. All participants were assessed at respective clinics between May 2018 and March 2020 by the same researcher (JK), mitigating potential assessor bias. All PwP were ambulant, and had been examined by a movement disorders neurologist for verification of the diagnosis in accordance with the UK Brain Bank Criteria for idiopathic PD (Hughes et al., 1992), and did not suffer from any other neurological disorder or from an inflammatory bowel disease. None had taken antibiotics in the 3-month period prior to collection of fecal samples. The study was approved by the Human Research Ethics Committees of the University of Western Australia (RA/4/20/4470) and St Vincent’s Hospital, Melbourne (LRR137/18), and was performed in accordance with the National Health and Medical Research Council guidelines and Good Clinical Practice Code. All study participants provided written informed consent and were free to withdraw from the study at any time.

### Clinical Assessment of PwP

A detailed history of gastrointestinal disorders; diet content; and antibiotic, probiotic, prebiotic, antacid or constipation treatment use within the previous year was obtained (Table 1). All study participants also completed a validated 15-question Gastrointestinal Symptom Rating Scale (GSRS) to evaluate the frequency and severity of gastrointestinal symptoms, as previously described (Kenna et al., 2021). All PwP were assessed in the “ON” state using the Movement Disorders Society Unified Parkinson’s Disease Rating Scale (MDS-UPDRS) Part III, and the Hoehn and Yahr scale, with a higher score and stage representing greater disease severity. Anti-parkinsonian medications were categorized into six main classes: levodopa (L-DOPA), dopamine agonists (DA), catechol-O-methyl transferase inhibitors (COMT-I), monoamine oxidase inhibitors (MAO-I), anticholinergics, and amantadine, and reported daily dosages were converted into a levodopa equivalent daily dosage (LEDD), as previously described (Bakeberg et al., 2019). The statistical testing of continuous and categorical variables was performed using ANOVA and the Fisher’s exact test, respectively, with *p* < 0.05 considered as statistically significant.

**TABLE 1:** Summary statistics of demographic and clinical assessment details.

Measure	PD (*n* = 87)	Controls (*n* = 47)
Age (years)	66.0 ± 9.1	61.7 ± 8.3
Male participants	55 (63.2%)	16 (34.0%)
Pro- or prebiotic use	16 (18.4%)	5 (10.6%)
Antacid use	27 (31%)	3 (6.4%)
Constipation medication use	45 (51.7%)	3 (6.4%)
GSRS total score	6.7 ± 4.2	4.6 ± 3.2
Disease duration in years	6.8 ± 5.4	–
Age of onset in years	59.2 ± 9.9	–
DBS	6 (6.9%)	–
LEDD (mg)	736.9 ± 568.1	–
Levodopa	73 (83.9%)	–
Dopamine agonists	38 (44.2%)	–
Catechol-o-methyl transferase inhibitors	17 (19.5%)	–
Monoamine oxidase inhibitors	25 (29.1%)	–
Amantadine	13 (14.9%)	–
Anticholinergics	0 (0%)	–
Unmedicated	4 (4.7%)	–
MDS-UPDRS part III^a^	23.4 ± 15.7	–
Motor fluctuations	41 (47.1%)	–
Hoehn and Yahr stage	2.1 ± 0.8	–

*Data are presented as *n* (%) or mean ± standard deviation. The superscript a indicates one missing value among the PwP samples, and thus the adjustment of case number for appropriate statistical measurements. PD, Parkinson’s disease; DBS, deep brain stimulation; LEDD, levodopa equivalent daily dosage; MDS-UPDRS, Movement Disorders Society unified Parkinson’s disease rating scale; GSRS, gastrointestinal symptom rating scale.*

### Stool Collection and DNA Extraction

Stool samples were self-collected at home by study participants using a provided sterile stool collection kit, with samples collected in polypropylene stool containers (P5744FS; Interpath, Victoria, Australia). Containers were stored briefly in a home freezer before being transported to a −80°C prior to DNA extraction. Fecal DNA extraction was performed using the phenol-chloroform-isoamyl method. Briefly, for each participant the entire fecal sample was homogenized with 70% (v/v) ethanol and a minimum of 20 g of fecal material centrifuged at 15,000rpm for 30 s. The resulting pellet was washed with TE buffer (500 μl; 10 mM Tris, 1 mM EDTA; Thermo Fisher, Scoresby, VIC, Australia), the supernatant removed, and the pellet combined with a mixture of TE buffer (200 μl), Buffer AL (200 μl; 19075; Qiagen, VIC, Australia) and Proteinase K (20 μl; 19131; Qiagen, VIC, Australia). Samples were then incubated at 56°C for 1 h, and an equal part ratio of the sample and phenol:chloroform:isoamyl 25:24:1 (CP0481; Rowe Scientific, WA, Australia) were added to a heavy phase lock gel tube (2302830, 5 PRIME Phase Lock Gel) and centrifuged for 4 min 15,000rpm. The resulting supernatant was processed again with phenol:chloroform:isoamyl, before being rinsed with chloroform:isoamyl 24:1 (CC5645; Rowe Scientific, WA, Australia). DNA was purified via standard ethanol precipitation protocol. In brief, two volumes of 95% (v/v) cold EtOH were combined with the supernatant, with the precipitated DNA being rinsed with 70% (v/v) EtOH and dissolved in DePC H_2_O (50 μL; 750023; Thermo Fisher, Scoresby, VIC, Australia) whilst being incubated at 56°C. The quality and quantity of DNA was examined using absorbance readings calculated by a NanoDrop One Microvolume UV-Vis spectrophotometer (Thermo Fisher Scientific Australia Pty LTD., Scoresby, VIC, Australia), with a minimum concentration of 10 ng/μL required.

### 16S Library Preparation

The V3V4 hypervariable region of the 16S rRNA gene was amplified using the S-D-Bact-0341-b-S-17 and S-D-Bact-0785-a-A-21 primer pair (Klindworth et al., 2013), and Illumina adapter overhang sequences were added to the 5′ ends of the forward and reverse primers, respectively (see Supplementary Table 2 for primer sequence details). The initial round of PCR amplification was performed in a 25 μL reaction mixture containing DNA (10 ng), *Taq* 2X Master Mix (12.5 μL; New England Biolabs, United States), forward and reverse primers (0.2 μM each), and diethyl pyrocarbonate (DEPC)-treated water (0.2 μM; Thermo Fisher Scientific, United States), under the following conditions: an initial denaturation cycle at 95°C for 30 s, 29 amplification cycles comprising denaturation (95°C for 30 s), annealing (50°C for 40 s), and extension (68°C for 1 min), and a final extension at 68°C for 5 min. PCR amplicons were examined via gel electrophoresis on a 1% (w/v) agarose gel, with appropriate controls to routinely assess for product purity and minimize contamination during this process. The PCR products were then purified using AMPure XP beads (Beckman Coulter, United States), and subjected to indexing PCR using the Nextera^®^ XT Index kit (Illumina, United States) according to manufacturer’s instructions. The libraries were sequenced on an Illumina MiSeq instrument using the MiSeq reagent kit v3 (600 cycles).

### Participant Sample Data Analysis

Acceptable DNA extraction and 16S rRNA sequencing were achieved for all 87 PwP and 47 control samples. Raw sequencing data were subjected to quality and adapter trimming via the bbduk.sh command available in BBTools^1^. The parameters used were: qtrim = r trimq = 20 ktrim = r k = 23 mink = 11 minlen = 200 hdist = 1 tpe tbo. Subsequently, the MeFit software was employed to merge overlapping paired-end reads with default parameters (Parikh et al., 2016). After merging, sequences with less than 420 bp were filtered. Clustering of the remaining sequences into operational taxonomic units (OTUs) with 99% sequence identity threshold using a *de novo* greedy algorithm, as well as the removal of chimeric sequences and singleton OTUs, were then performed via the *micca otu* command available in Micca software (version 1.7.2) (Albanese et al., 2015). Taxonomic classification of each representative OTU sequence was performed using the Bayesian LCA-based taxonomic classification method against the NCBI 16S ribosomal RNA database (Gao et al., 2017), following which a minimum confidence score of 80 was used to accept a taxonomic assignment at each level.

Following rarefaction of the OTU table at 16625 sequences per sample, both alpha and beta diversities were analyzed using the phyloseq (McMurdie and Holmes, 2013) and microbiomeSeq^2^ R packages. For alpha diversity analysis, the species richness and Shannon diversity metrics were employed, and the group differences were compared using ANOVA. For beta diversity analysis, principal coordinates analysis (PCoA) was performed at the OTU level using both weighted and unweighted UniFrac distance metrics, followed by the analysis of similarity test (ANOSIM) to test the significance of the difference between groups. Additionally, functional prediction of the microbial communities for KEGG pathways was performed using PICRUSt2 (version 2.3.0-b) (Douglas et al., 2020). Specific R codes used to generate these analyses are available in Supplementary File S3.

### Identification of Significant Taxa and KEGG Pathways

Differential abundance analysis was performed on bacterial taxa and KEGG pathways that were each present in at least 15% of the total samples, and with a minimum mean relative abundance of 0.01%. The *Wilcoxon rank*-*sum* test was employed and then followed by false discovery rate adjustment using Storey’s *q*-value approach (Storey, 2002). Taxa and KEGG pathway abundance differences with *q* < 0.15 and *q* < 0.05, respectively, were considered statistically significant.

## Results

### Demographic and Clinical Data

The demographic and clinical assessment data are summarized in Table 1. Overall, a higher proportion of PwP were male compared to the participants without PD (henceforth referred to as control participants: 63.2 vs. 34.0%, *p* = 0.001), and the average age of PwP was four years older than that of the control subjects (66.0 ± 9.1 vs. 61.7 ± 8.3, *p* = 0.008). The prevalence of constipation medication and antacid use were also significantly greater among the PwP cohort than in the controls (constipation: 51.7 vs. 6.4%, *p* < 0.001; antacid use: 31 vs. 6.4%, *p* < 0.001). When estimating the severity of gastrointestinal symptoms, PwP had a greater GI symptom burden (represented by higher GSRS scores) as compared to the controls (6.7 ± 4.2 vs. 4.6 ± 3.2, *p* = 0.003). Within the PwP cohort, participants had a mean MDS-UPDRS III score of 23.4 ± 15.7, and Hoehn and Yahr scores ranged from stage 1–5, with 19.5, 62.1, 13.8, 3.4, and 1.1% falling into each stage respectively. Almost half of the PwP (47.1%) experienced motor fluctuations or time in the “off state.” The majority of PwP were taking anti-parkinsonian medications (95.3%), with levodopa being the most common medication (83.9%). None were being treated with levodopa–carbidopa intestinal gel or anticholinergics.

### Diversity Analysis of the Gut Microbiota of PwP and Control Subjects

A total of 7,492,605 reads were generated for the 87 PwP and 47 control subjects. Following quality trimming and merging of overlapping paired-end reads, 4,313,118 sequences remained, ranging from 20,234 to 83,632 per sample with an average read length of 446 ± 5 bp (Supplementary Table 1). Taxonomic classification of the 11,521 OTUs generated by *de novo* sequence clustering at 99% identity threshold revealed 203 genera, 64 families, 35 orders, 22 classes and 10 phyla.

**FIGURE 1:**
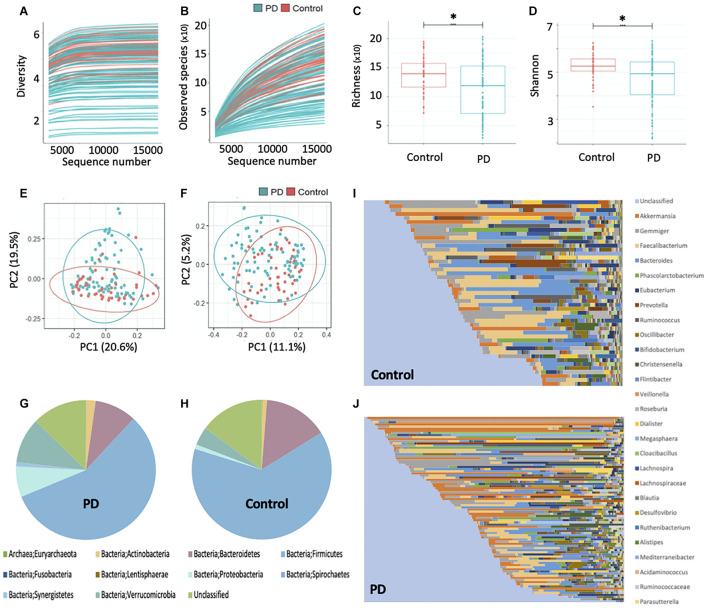
Gut microbiota sequencing overview in controls and PwP. **(A)** Rarefaction curve based on Shannon diversity index. **(B)** Rarefaction curve based on species richness index. **(C)** Comparison of alpha diversity based on species richness index and **(D)** Shannon diversity index, between PwP and control cohorts using ANOVA. **(E)** Principal coordinates analysis based on weighted and **(F)** unweighted UniFrac distance metrics. **(G)** Relative abundance of bacterial phyla in PwP and **(H)** control cohorts. **(I)** Relative abundance of bacterial genera in control and **(J)** PwP cohorts. Relative abundance was calculated using OTU values, and each color represents a different bacterial taxon. ^∗^ indicates *p* < 0.001. Figures were created using R v. 4.0.4 and Microsoft Excel.

The plateauing rarefaction curves (Figures 1A,B) based on Shannon diversity and species richness indices, respectively, indicate that there is sufficient coverage of species richness and microbial diversity per sample. Alpha diversity analysis further demonstrated that the gut microbial richness and diversity of the PwP cohort was significantly less than that of the controls (*p* < 0.001 for both species richness and Shannon diversity metrics; Figures 1C,D). The beta-diversity analysis, however, showed no separation between the PwP and control samples on the PCoA plots (Figures 1E,F). Further testing using ANOSIM revealed that the microbial community composition of the two groups was similar (unweighted UniFrac: *R* = −0.025 and *p* = 0.769; weighted UniFrac: *R* = −0.044 and *p* = 0.940).

### The Difference in Gut Microbiota Composition Between PwP and Control Participants

**FIGURE 2:**
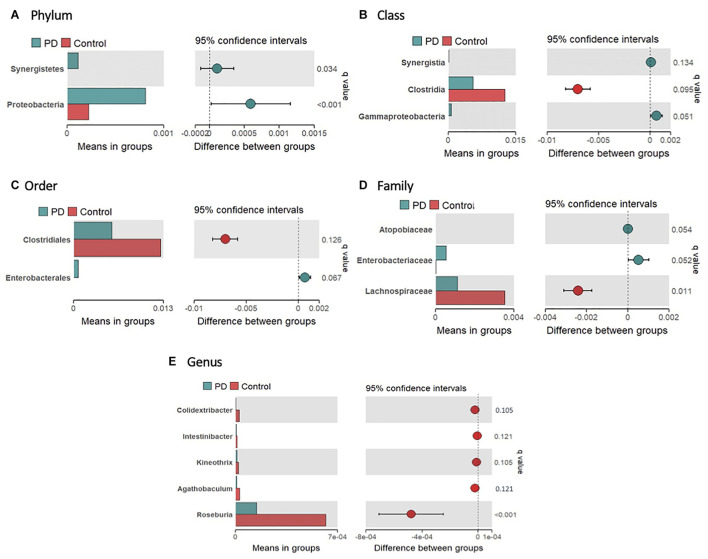
Significantly different taxa in the gut microbiome of controls and PwP following 16S rRNA sequencing. Relative abundance of significantly different bacteria at phylum **(A)**, class **(B)**, order **(C)**, family **(D)**, and genus **(E)** levels between controls (red) and PwP (blue). Only bacteria with significant differences are presented. The bars on the left side of each figure show mean relative abundance between PwP and controls. The figures to the right represent the difference between means of PwP and control participants, with error bars representing the 95% confidence interval. *Q*-values are presented above each individual taxon and were generated using Wilcoxon rank-sum test followed by FDR adjustment with Storey’s *q*-value. *q* < 0.15 was considered statistically significant. Figures were created using R v. 4.0.4.

The average gut microbial composition of the PwP and control cohorts at the phyla level are depicted in Figures 1G,H, and at the genus level in Figures 1I,J, respectively. The three most abundant phyla detected in the PwP cohort were Firmicutes (56.8%), Verrucomicrobia (10.5%) and Bacteroidetes (9.7%), whereas in the control cohort they were Firmicutes (63.6%), Bacteroidetes (14.9%) and Verrucomicrobia (4.3%). The three most abundant genera detected in the PD cohort were *Akkermansia* (10.5%), *Gemmiger* (7.4%) and *Faecalibacterium* (7.0%), in comparison to controls with *Faecalibacterium* (10.6%), *Bacteroides* (10.1%), and *Gemmiger* (7.0%). To analyze potential differences in the composition of the gut microbiota in PwP compared to controls we calculated the relative abundances of individual taxa at different phylogenetic ranks and found significant differences in two phyla, three classes, two orders, three families and five genera (Figures 2A–E). Specifically, our samples indicated significantly increased Proteobacteria (*q* = 0.034) [Gammaproteobacteria (*q* = 0.051); Enterobacterales (*q* = 0.067); *Enterobacteriaceae* (*q* = 0.052)] and Synergistetes (*q* < 0.001) [Synergistia (*q* = 0.134)], and significantly decreased Clostridia (*q* = 0.095) [Clostridiales (*q* = 0.126); *Lachnospiraceae* (*q* = 0.011); *Roseburia* (*q* < 0.001), *Kineothrix* (*q* = 0.105), *Agathobaculum* (*q* = 0.121), *Colidextribacter* (*q* = 0.105), and *Intestinibacter* (*q* = 0.121)] in PwP when compared to controls. We did not analyze the species differences, as 16S rRNA sequencing is not a reliable indicator of species abundance (Johnson et al., 2019).

### Microbial Associations With Gender, Constipation and Use of GI Medications

When the PwP cohort was split by gender there were no significant differences or groupings in regard to the microbiome composition. In addition, as it has been reported that chronic constipation *per se* may be associated with changes in the gut microbiota (Zhao and Yu, 2016), the PwP subgroup who were constipated were compared with those not, and analysis showed no significant differences in bacterial taxa between the two groups. Similarly, no significant differences were observed in those who regularly used antacid medications or pro- or prebiotics.

### Functional Analysis of the Gut Microbiota

**FIGURE 3:**
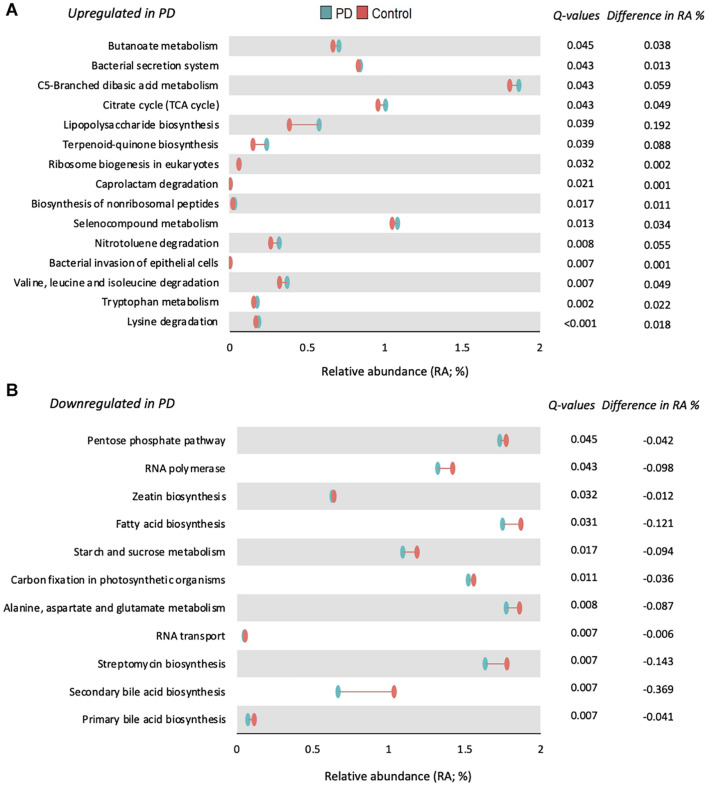
Functional prediction of 26 significantly altered KEGG pathways in PwP. Differences in relative abundance of metabolite pathways predicted to be significantly upregulated **(A)** or downregulated **(B)** in PwP (blue) compared to controls (red). *Q*-values are presented to the right of each pathway and were generated using Wilcoxon rank-sum test followed by FDR adjustment with Storey’s *q*-value. The difference in median relative abundance between PwP and control participants is also presented. *q* < 0.05 was considered statistically significant. Figures were created using R v. 4.0.4.

Functional prediction of the microbial communities present among our samples revealed 165 KEGG pathways, following which 124 with a mean relative abundance of at least 0.01%, and a prevalence of least 15% across the entire samples were subjected to statistical comparison. As depicted in Figure 3, 26 KEGG pathways were significantly altered in PwP, when compared to healthy controls: 15 up-regulated (Figure 3A) and 11 down-regulated (Figure 3B). It is important to note that lysine degradation (*q* < 0.001), tryptophan metabolism (*q* = 0.002), and valine, leucine, and isoleucine degradation (*q* = 0.007) were the three most up-regulated pathways, whereas primary and secondary bile acid biosynthesis (*q* = 0.007) was among the most down-regulated pathways in our PwP.

## Discussion

In view of the known geographic influences on the composition of the gut microbiome, there is an important need to explore this to further understanding of the role of the gut microbiome in Parkinson’s disease (PD). Previous studies investigating the relationship between the gut microbiome and PD have not comprehensively investigated populations in the southern hemisphere, thus it was unknown whether PwP had differences in their gut microbiome similar to that reported in existing literature. In this study we used 16S rRNA sequencing followed by KEGG analysis to investigate the differences in the gut microbiota and resulting changes in metabolic pathways between PwP and controls. Our findings demonstrate that the gut microbiota, and 26 associated metabolic pathways, are significantly altered in Australian PwP compared to non-affected individuals.

Firstly, alpha diversity analysis indicated a reduced richness and diversity within the PwP cohort in comparison to the control cohort. A reduced alpha diversity has also been reported in Alzheimer’s Disease (Vogt et al., 2017), gastrointestinal disorders (Saffouri et al., 2019), and obesity (Stanislawski et al., 2019). The overall microbiome composition of both PwP and controls were predominated by Firmicutes (Controls: 63.6%, PwP: 56.8%), which is reported frequently in individuals who consume a Western diet (Nandipati and Litvan, 2016) such as that in Australia. As noted in our previous preliminary study, the abundance of the phylum Verrucomicrobia was greater than double in PwP (Controls: 4.25 vs. PwP: 10.5%), which has been reported to populate a healthy individual’s microbiota from 1–5% (Roy, 2017). Importantly, Verrucomicrobia are mucin-degrading bacteria (Gorecki et al., 2019), thus it is plausible that their dominance could contribute to an increase in intestinal permeability and inflammation. Significant bacterial differences were identified more broadly in two phyla, three classes, two orders, three families, and five genera in PwP when compared to non-affected individuals (see Figure 2). In the present study we report comparable findings to previous studies from the Northern hemisphere in regard to changes in gut microbiota composition, with increases in the pro-inflammatory bacterial phyla Proteobacteria (Gammaproteobacteria, Enterobacterales, and *Enterobacteriaceae*), and decreases in commensal bacteria Clostridia, from the Firmicutes phylum (Keshavarzian et al., 2015; Scheperjans et al., 2015; Sampson et al., 2016; Unger et al., 2016; Hill-Burns et al., 2017; Hopfner et al., 2017; Li et al., 2017; Petrov et al., 2017; Heintz-Buschart et al., 2018; Qian et al., 2018; Aho et al., 2019; Gorecki et al., 2019; Li et al., 2019; Pietrucci et al., 2019; Weis et al., 2019). Within our results, *Lachnospiraceae* and its related genera were decreased in PwP. This bacterial family is generally considered a significant producer of anti-inflammatory short chain fatty acids (SCFA) and are associated with a healthy functioning gut (Wong et al., 2006). Low levels of the *Lachnospiraceae* family have also been reported previously in individuals with diarrhea, Crohn’s disease and ulcerative colitis (Flint et al., 2012; Donaldson et al., 2016). Of particular interest are the genera *Roseburia* and *Agathobaculum* which were also both significantly reduced in our PwP cohort. Both of these bacterial genera are producers of the SCFA butyrate that has demonstrated protective effects on dopaminergic cell death, and reduced motor impairments and dopamine deficiency when administered in animal models of PD (St Laurent et al., 2013; Paiva et al., 2017; Getachew et al., 2020). Furthermore, butyrate also regulates the permeability of the gut through the stimulation of tight junctions and mucus production (Getachew et al., 2020), and promotes an acidic gut pH which reduces the growth of pathogenic pro-inflammatory bacteria (Walker et al., 2005). The reduction in *Agathobaculum*, which exhibits effects on gut motility regulating ability, could be a contributory factor to the increased severity and frequency of GI dysmotility symptoms in this particular cohort (Kenna et al., 2021), as well as other PwP cohorts (Kaye et al., 2006; Knudsen et al., 2017; Li et al., 2019; Cirstea et al., 2020). On the other hand, Gammaproteobacteria, a pro-inflammatory bacterium that produces endotoxins and is associated with GI disorders such as Crohn’s disease (Sekido et al., 2020), and Synergistetes (Synergistia) a bacterium involved in GI infections (Vartoukian et al., 2007), were significantly increased in our PwP cohort. Together, these findings support a significantly altered, pro-inflammatory gut microbiome in PwP.

The functional implications of such changes within the gut microbiome between diseased and non-affected control populations have not been adequately investigated and remain a significant challenge within the field of PD and wider microbiome research. To this end, in the present study we combined the compositional 16S microbiota analysis with computational KEGG modeling to gain insight into the functional effects of the changed microbial abundances in PwP, and their significance in understanding the interaction between the gut microbiome and PD. Of the 26 significantly altered pathways (see Figure 3), two downregulated pathways were involved in primary and secondary bile acid biosynthesis. Primary bile acids are synthesized in the liver and secondary bile acids are synthesized in the colon as a result of microbial activity (Ridlon et al., 2016). Bile acids eliminate cholesterol from the body (Ticho et al., 2019), aid GI motility (Ticho et al., 2019), and reduce the bacteria found in the small intestine (Ridlon et al., 2014). This corresponds with previous research indicating constipation as one of the first symptoms in PD and identifying that PwP are more likely to be constipated than the general public (Kenna et al., 2021). Another interesting implication of this finding would help explain the increased prevalence of small intestinal bacterial overgrowth (SIBO) in PwP, as the reduced biosynthesis of bile acids would diminish the capacity to moderate bacterial populations in the small intestine. In relation to the downregulation of secondary bile acid biosynthesis in PwP, it has been suggested that this pathway is dependent on a “microbial community” (Schmidt et al., 2010), thus we postulate that this may be ensuing from the reduced gut microbial diversity demonstrated in this cohort of PwP. Although not conclusive, recent studies suggest a loss of HDL cholesterol is associated with an increased inflammatory response and increased risk of developing neurodegenerative disorders like PD (Pes et al., 2021). Although we did not measure HDL levels within our cohort, it presents as a promising area of interest for future exploration. Furthermore, as previous studies have suggested that bile acids are protective in animal models of neurodegeneration, protecting against mitochondrial damage specifically in PD (Abdelkader et al., 2016), and having other effects in Alzheimer’s disease (Nunes et al., 2012) and amyotrophic lateral sclerosis (Elia et al., 2016) models, this mechanism warrants further investigation in relation to its role in PD pathophysiology.

Seven of the significantly altered metabolic pathways involve amino acid degradation or metabolism, and ones of particular interest were alanine, valine and tryptophan. Overall, amino acid biosynthesis was predicted to decrease and metabolism to increase. Defects in the metabolism of amino acids valine, leucine or isoleucine can produce motor dysfunction and neurodegeneration (Mor et al., 2020), recapitulating major features of PD, and suggesting that normal amino acid metabolism is critical for normal physiological and biological functioning. Environmental alanine exposure has been associated with neurodegenerative disorders, however, this hypothesis does require further verification (Figura et al., 2018). Alanine has also been shown to break down tryptophan, thus the exhibited decrease in metabolism of alanine corresponds with the demonstrated increase in metabolism of tryptophan within our cohort of PwP. Although we did not see an association between PD medication and the gut microbiota in our cohort, it is important to note that literature is currently investigating the possible effect levodopa has on decreasing tryptophan metabolism via the serotonergic system (Stansley and Yamamoto, 2015), therefore highlighting this as something to consider in further studies.

Accelerated tryptophan metabolism has commonly been considered as a feature of clinical ailments such as inflammation, infection and malignant disease (Gostner et al., 2020), especially IBD (Nikolaus et al., 2017), which as we have previously discussed also increases the risk of developing PD (Lai et al., 2019; Villumsen et al., 2019). Metabolites of tryptophan metabolism are hypothesized to extensively influence systemic inflammation as well as blood brain barrier permeability (Szabo et al., 2011). These results align with previous studies reporting increased catabolites of tryptophan in PwP which related to disturbances in mitochondrial function and brain energy metabolism involved in the development of neurodegenerative diseases (Szabo et al., 2011; Luan et al., 2015). Tryptophan metabolism is also involved with the formation of neurotransmitters such as serotonin and melatonin. IDO-1 is a tryptophan degrading enzyme which recognizes serotonin as a substrate, and the predicted increase in tryptophan metabolism may suggest increased IDO-1 activity. Interestingly, increased IDO-1 activity has been reported to contribute to mood-lowering (Gostner et al., 2020), which is a common comorbidity reported in PwP. Fittingly, 95% of serotonin is synthesized and stored in the GI tract, where it modulates GI secretion and motility. Thus, an increase in IDO-1 activity may reduce the amount of serotonin in the gut, possibly underlying the decreased motility previously reported in this cohort of PwP (Kenna et al., 2021). Melatonin is also a by-product of the serotonin pathway, and as it is predicted that serotonin levels are depleted in PwP, the increased prevalence of sleep disorders in PwP (Suzuki et al., 2011) could be due to a decreased level of melatonin. It is also important to mention that as aging is associated with decreased tryptophan concentration (Capuron et al., 2014), it could be a commonality among an older population. However, as our analyses controlled for age, this potential variable has been mitigated in this study. Nevertheless, amino acids and their associated metabolic pathways present as a promising target for future therapeutic research in PD.

For decades, exposure to environmental toxins has been a proposed risk factor for the development of PD (Nandipati and Litvan, 2016). Accordingly, three pathways involved in toxin degradation were predicted to be significantly increased in PwP in this study: Caprolactam degradation, Selenocompound metabolism, and Nitrotoluene degradation. Despite selenium being an essential nutritional element, excessive levels cause toxicity and adverse effects (Rayman, 2020), with previous studies demonstrating links between elevated selenium levels in PwP and increased PD mortality (Ellwanger et al., 2016; Adani et al., 2020). Another compound of interest is nitrotoluene which is primarily used in dyes for cotton, wool, silk, leather and paper, and the synthesis of explosives, organic chemicals, pesticides, petrochemicals and pharmaceuticals [National Toxicology Program (NTP), 2016], exposure to which can detrimentally modify the microbiome into a pro-inflammatory state. Both compounds are indicated as human carcinogens, thus exposure can be very detrimental to health. Interestingly, literature suggests that GI microbial composition plays an obligatory role in toxin degradation and toxicity (Claus et al., 2016), thus it is plausible that individuals who develop PD may be predisposed to a gut microbiome that has reduced capacity to degrade toxins, or one that is predisposed to succumb more rapidly to toxicity.

Finally, three pathways that were predicted to be upregulated in PwP are known to be involved in the virulence of pathogenic bacteria. The biosynthesis of “siderophore group NRPS,” which is involved in ferritin acquisition for growth and proliferation, has been shown to be a crucial factor in determining the virulence of many pathogenic bacterial and fungal species (Carroll and Moore, 2018). In addition, the “bacterial secretion system” pathway is responsible for secreting virulence factors into the host; and the “bacterial invasion of epithelial cells” pathway, whereby pathogenic bacteria induce colonization, followed by dissemination into other cells (Ribet and Cossart, 2015). Together, the upregulation of these pathways is compatible with a cycle in which pathogenic bacteria are undergoing proliferation and dissemination, and thus, may explain the dysbiotic microbiome evident in PwP.

## Future Directions and Conclusion

The current study provides the first comprehensive characterization of the gut microbiome in Australian PwP. It is important to note, a limitation of this study is the small proportion of drug-naïve PwP in this cohort, and thus the inability for further group comparisons to exclude the effects of drug treatment. In addition, the temporal changes within the gut microbiota could not be considered in this cross-sectional study, which requires a longitudinal study design allowing for the effects of confounding variables (such as diet and exercise). While it is clear that additional studies are required to elucidate the complex role of the gut microbiota and how they influence host functional pathways in PwP, the results of the present study illuminate a number of areas of interest that present as promising targets to be further explored. Firstly, as many of the pathways which are predicted to be significantly affected in PwP have an involvement with the immune system, such as adverse effects of low lysine levels on the production of immune cells and the role of alanine in strengthening the immune system, it would be of interest to investigate the relationship between changes in the gut microbiome and various immune components at different stages of disease progression in future studies. Secondly, as bacteria are not the only constituents of the gut microbiome, and the fungi, viruses and protozoa in the gut microbiome are known to influence the bacterial composition of the gut microbiota, it will be important to investigate the changes in these other components of the gut microbial “ecosystem” in PD. This is an area of human gut microbiome research that is currently understudied, with only a single study to date looking at 18S fungal sequencing in PD (Heintz-Buschart et al., 2018). Lastly, to better understand the interaction between factors underlying PD pathophysiology, integrating various approaches such as microbiome and dietary analysis, metabolomics and computational biology approaches is likely to help identify key interactions in an unbiased manner. Such approaches could also be individualized to determine the effects of different anti- parkinsonian medications, and possibly to help predict optimal treatment routes and treatment responses. As the prevalence of PD is rapidly rising (Rocca, 2018), investigations into the pathophysiological underpinnings of the disease are increasing in demand, and findings are urgently required.

## Data Availability Statement

The datasets presented in this study can be found in online repositories. The names of the repository/repositories and accession number(s) can be found below: https://figshare.com/, https://doi.org/10.6084/m9.figshare.14345513.

## Ethics Statement

The studies involving human participants were reviewed and approved by The University of Western Australia Human Research Ethics Committee RA/4/20/4470, and St Vincent’s Hospital Melbourne Human Research Ethics Committee LRR137/18. The patients/participants provided their written informed consent to participate in this study.

## Author Contributions

JK: conception, organization, and execution of the research project, design, execution, and review and critique of the statistical analysis, and writing of the first draft and review and critique of manuscript preparation. EC: design and execution of statistical analysis and writing of the first draft and review and critique of manuscript preparation. MB: execution of research project, review and critique of statistical analysis, and review and critique of the manuscript preparation. AT: organization and execution of research project and review and critique of manuscript preparation. SM: organization and execution of research project. AG: review and critique of statistical analysis and review and critique of manuscript preparation. MH: organization and execution of research project and review and critique of manuscript preparation. BM: organization and execution of research project. FM: conception of research project, review and critique of statistical analysis, and writing of the first draft and review and critique of manuscript preparation. RA: conception and organization of research project, design, execution, review and critique of statistical analysis, and writing of the first draft and review and critique of manuscript preparation. All authors contributed to the article and approved the submitted version.

## Conflict of Interest

The authors declare that the research was conducted in the absence of any commercial or financial relationships that could be construed as a potential conflict of interest.

## Publisher’s Note

All claims expressed in this article are solely those of the authors and do not necessarily represent those of their affiliated organizations, or those of the publisher, the editors and the reviewers. Any product that may be evaluated in this article, or claim that may be made by its manufacturer, is not guaranteed or endorsed by the publisher.

## Footnotes

1
https://jgi.doe.gov/data-and-tools/bbtools/

2
https://github.com/umerijaz/microbiomeSeq

## Supplementary Material

Supplementary Table 1 Raw sequencing data following V3 V4 16s rRNA analysis.

Supplementary Table 2 Primer sequences for PCR.

## Abbreviations

GI: Gastrointestinal
IBD: Inflammatory Bowel Disease
KEGG: Kyoto Encyclopedia of Genes and Genomes
PCR: Polymerase chain reaction
PD: Parkinson’s disease
PwP: People with Parkinson’s disease
rRNA: Ribosomal ribonucleic acid.
