# Supplementary material for: Changes in the Gut Microbiome and Predicted Functional Metabolic Effects in an Australian Parkinson’s Disease Cohort
Source: Front Neurosci. 2021 Oct 29;15:756951. doi: 10.3389/fnins.2021.756951 (PMC8588830; doi:10.3389/fnins.2021.756951)
Supplement: Supplementary file 1 [file Data_Sheet_1.DOCX]

**Supplementary file S3. R codes for participant sample analysis.**

library("phyloseq")

library("ggplot2")

library("amplicon")

library("microbiomeSeq")

library("ape")

otu <- as.matrix(read.table("otutable_rare.txt"))

tax <- as.matrix(read.table("taxa_BLCA.csv"))

tree <- read.tree("msa_rooted.tree")

map <- read.csv("Metadata.csv", row.names=1)

phyloseq <- phyloseq(otu_table(otu, taxa_are_rows = TRUE),tax_table(tax),phy_tree(tree),sample_data(map))

rare1 = alpha_rare_all(ps = phyloseq, group = "Group", method = "diversity_shannon", start = 500, step = 500)

p1<-rare1[[1]]

ggsave("Rarefaction_curve_shannon.tiff", units = c("in"), width=6, height=4, dpi=300, compression="lzw", scale=1, p1)

rare2 = alpha_rare_all(ps = phyloseq, group = "Group", method = "observed", start = 500, step = 500)

p2<-rare2[[1]]

ggsave("Rarefaction_curve_richness.tiff", units = c("in"), width=6, height=4, dpi=300, compression="lzw", scale=1, p2)

plot_anova_diversity(phyloseq, method = c("richness", "shannon"), grouping_column = "Group", pValueCutoff = 0.05, outfile = "alphadiv_ANOVA_group.csv")

ggsave("alphadiv_ANOVA_group.tiff", units = c("in"), width=15, height=10, dpi=300, compression="lzw")

ord.wunifrac<-ordinate(phyloseq, method = "PCoA", distance = "wunifrac")

p6<-plot_ordination(phyloseq, ord.wunifrac, color = "Group") + theme_bw() + ggtitle("Weighted UniFrac distance") + stat_ellipse(aes(group = Group))

ggsave("betadiv_Group_weighted_UniFrac.tiff", units = c("in"), width=8, height=6, dpi=300, compression="lzw", scale=1, p6)

ord.uunifrac<-ordinate(phyloseq, method = "PCoA", distance = "uunifrac")

p7<-plot_ordination(phyloseq, ord.uunifrac, color = "Group") + theme_bw() + ggtitle("Unweighted UniFrac distance") + stat_ellipse(aes(group = Group))

ggsave("betadiv_Group_unweighted_UniFrac.tiff", units = c("in"), width=8, height=6, dpi=300, compression="lzw", scale=1, p7)
