# Supplementary material for: Changes in the Gut Microbiome and Predicted Functional Metabolic Effects in an Australian Parkinson’s Disease Cohort
Source: Front Neurosci. 2021 Oct 29;15:756951. doi: 10.3389/fnins.2021.756951 (PMC8588830; doi:10.3389/fnins.2021.756951)
Supplement: Supplementary Table 2 — Primer sequences for PCR. [file Table_2.docx]

**Supplementary Table S2.** Primer Sequences for PCR.

| Primer name | Sequence |
| --- | --- |
| 16S Amplicon PCR Forward Primer | 5'-TCGTCGGCAGCGTCAGATGTGTATAAGAGACAGCCT  ACGGGNGGCWGCAG-3’ |
| 16S Amplicon PCR Reverse Primer | 5'GTCTCGTGGGCTCGGAGATGTGTATAAGAGACAGGA  CTACHVGGGTATCTAATCC-3’ |
| Forward primer overhang | 5’-TCGTCGGCAGCGTCAGATGTGTATAAGAGACAG-3’ |
| Reverse primer overhang | 5’-GTCTCGTGGGCTCGGAGATGTGTATAAGAGACAG-3’ |
